# Coulomb interactions in high-coherence femtosecond electron pulses from tip emitters

**DOI:** 10.1063/1.5066093

**Published:** 2019-01-30

**Authors:** Nora Bach, Till Domröse, Armin Feist, Thomas Rittmann, Stefanie Strauch, Claus Ropers, Sascha Schäfer

**Affiliations:** 1 4th Physical Institute - Solids and Nanostructures, University of Goettingen, Goettingen, Germany; 2 Institute of Physics, University of Oldenburg, Oldenburg, Germany

## Abstract

Tip-based photoemission electron sources offer unique properties for ultrafast imaging, diffraction, and spectroscopy experiments with highly coherent few-electron pulses. Extending this approach to increased bunch-charges requires a comprehensive experimental study on Coulomb interactions in nanoscale electron pulses and their impact on beam quality. For a laser-driven Schottky field emitter, we assess the transverse and longitudinal electron pulse properties in an ultrafast transmission electron microscope at a high photoemission current density. A quantitative characterization of electron beam emittance, pulse duration, spectral bandwidth, and chirp is performed. Due to the cathode geometry, Coulomb interactions in the pulse predominantly occur in the direct vicinity to the tip apex, resulting in a well-defined pulse chirp and limited emittance growth. Strategies for optimizing electron source parameters are identified, enabling advanced ultrafast transmission electron microscopy approaches, such as phase-resolved imaging and holography.

## I. INTRODUCTION

The observation of ultrafast nanoscale dynamics promises a profound understanding of the spatio-temporal dynamics of elementary excitations in solids and thus new avenues for tailoring and controlling the flow of energy, charges, and spins in nanostructured materials. Experimental methods to access processes on femtosecond time and nanometer length scales are highly desired. Substantial progress has recently been achieved employing ultrafast optical near-field techniques,^1–4^ terahertz scanning tunneling microscopy,^5,6^ and pump-probe approaches with x-ray^7–9^ or electron pulses.^10,11^ Due to their large scattering cross-section and their intrinsically short wavelength, electrons are a natural choice for ultrafast nanoscale imaging. In particular, ultrafast electron microscopy (UEM)^12–16^ and diffraction (UED)^11,17–19^ have provided a rich picture of femtosecond and picosecond processes such as optically triggered phase transitions in correlated materials^20–23^ and phonon dissipation^19,24–29^ in nanostructures. The spatio-temporal resolution in these approaches crucially depends on the transverse and longitudinal electron pulse characteristics, including pulse duration, spatial coherence, and bunch charge. In UED-type experiments, few nm-scale transverse coherence lengths are often sufficient to resolve the temporal dynamics in diffraction patterns of materials with a small unit cell.^19^ A higher degree of spatial coherence becomes important for resolving the dynamics related to larger unit cells, detailed spot profile analyses, and phase ordering phenomena.^23,30,31^ For ultrafast electron imaging at nanometer length scales, a central figure-of-merit is the beam brightness, which is directly proportional to the electron current per occupied phase space area.^32^ The beam brightness can be enhanced by reducing the photoemission excess energy, achievable by optimized photocathode materials.^33–35^ In addition, minimizing the source size has a significant impact on the brightness and, in the past, was indispensable for the development of state-of-the-art continuous high-brightness electron sources, such as Schottky^36,37^ and cold field emitters,^38^ as well as single- and few-atom sources.^39,40^ The concept of tip-shaped electron sources has therefore been adopted for pulsed photoemitters with nm-sized emission areas.^16,41–48^ The increased coherence properties of such tip-emitted electron pulses and their application for locally probing ultrafast phenomena were recently demonstrated for the quantum coherent optical control of free-electron states^49^ and the nanoscale mapping of ultrafast structural^25^ and magnetic dynamics.^50^

For dense electron beams and multi-electron pulses, Coulomb interactions constrain the beam quality in both the longitudinal and transverse directions. Specifically, the mean Coulomb field induces a reversible deformation of the phase space distribution, whereas stochastic (pulse-to-pulse) local charge fluctuations irreversibly spread the ensemble-averaged phase space distribution. This phenomenon is described as stochastic trajectory displacements^51^ for the transverse direction and termed the Boersch effect^52^ for the energy broadening corresponding to the longitudinal direction.

During the last few decades, brightness limitations caused by Coulomb interactions have been the subject of many experimental^18,34,53,54^ and theoretical studies^55–64^ considering continuous electron sources as well as pulsed photoemitters.

Reversing the mean-field induced linear chirp of electron pulses is an important topic in ultrafast electron imaging, diffraction, and spectroscopy. Coherent phase space manipulation via deceleration and acceleration of electrons in oscillating radio-frequency cavities^54,65^ or by using terahertz fields^66,67^ has resulted in temporal pulse durations down to a few femtoseconds.

Despite the large body of work on Coulomb interactions in ultrashort electron pulses, little is known on their impact on pulses derived from nanoscale emitters.^62,68^ In particular, the small emission area, the large beam divergence angle, and strong extraction fields at nanotips suggest space-charge behaviors distinctively different from flat photocathode emission. A further brightness improvement of laser-driven tip emitters calls for a systematic study.

In this work, we demonstrate the application of tip-shaped photocathodes in a regime for which high photocurrent densities are formed in the vicinity of the emitter. Quantitative characterization of the longitudinal and transverse electron beam properties allows for identifying different operation modes of the photoemitter, optimized for high temporal or high spatial resolution. A simplified numerical model is presented to describe the experimental dependence of the transverse beam coherence, spectral bandwidth, and temporal chirp on the bunch charge.

## II. SETUP

**FIG. 1.**
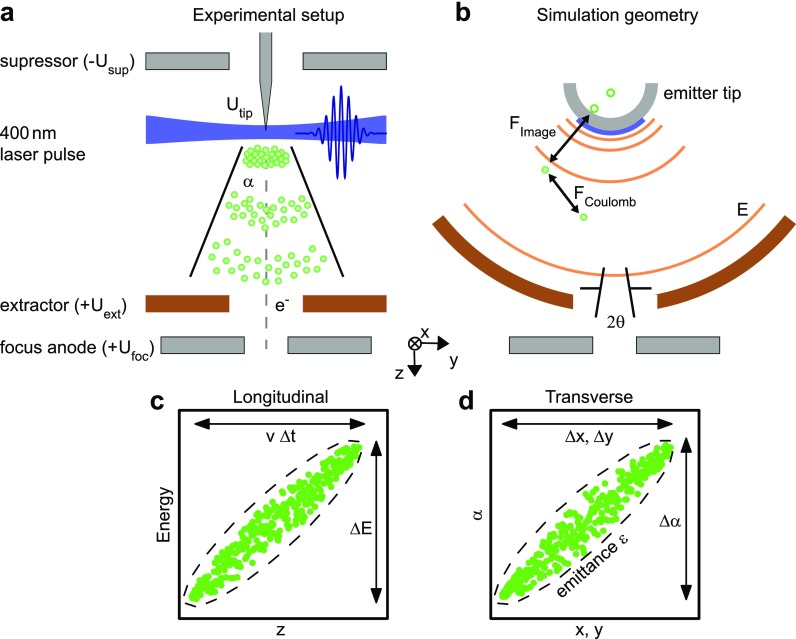
Experimental and simulation geometry and phase space representations. (a) Experimental electrode assembly with the Schottky-type ZrO/W field emission tip. Electrostatic potentials are given relative to the potential of the emitter. (b) Simulation geometry based on a spherical capacitor model. Propagation of the electron pulse considers the acceleration in the external electrostatic field; Coulomb interaction of the electrons within the pulse and with image charges at the emitter. (c) and (d) Sketched electron distribution (green dots) in the phase spaces corresponding to the longitudinal (c) and transverse directions (d). Longitudinal and transverse pulse properties are characterized by the energy spread and pulse duration and angular and spatial width, respectively. The transverse beam quality is given by the beam emittance, which is related to the occupied phase-space area.

The Göttingen ultrafast transmission electron microscope (UTEM) is based on a Schottky field-emission JEOL JEM-2100F TEM instrument, which we modified to allow for a laser-triggered photoelectron mode and for optical sample excitation.^16^ Femtosecond electron pulses are generated by employing localized single-photon photoemission from the apex of a Schottky-type ZrO/W field emission tip which is placed into an electrostatic electrode assembly [Fig. 1(a)]. The photoemitter is side-illuminated with 400-nm laser pulses focused to a spot diameter of about 20 *μ*m (full-width-at-half-maximum, FWHM) at a 250-kHz repetition rate. The electron bunch charge and pulse duration are varied by the laser intensity and pulse duration. The acceleration field at the tip apex is governed by the applied electrostatic potentials at the extractor ($U_{\text{ext}}$) and suppressor ($U_{\text{sup}}$) electrodes. At the focus electrode, the central section of the electron beam is filtered by an aperture with an effective size depending on the electrode potentials. For example, a transmission ratio through the focus electrode in the range of 0.1%–1% is expected for potentials of $U_{\text{ext}}=1\;\text{kV}$ and $U_{\text{sup}}=-300\;V$ (relative to the emitter).

Behind the focus anode, the electron pulses are accelerated to an electron energy of up to 200 keV (here operated at 120 keV) and coupled into the electron optics of a TEM column (condenser aperture: 100 *μ*m, transmission ratio about 25%). Electron beam caustics are recorded by through-focus series around the sample plane. The minimum electron spot diameter and angular spread in the sample plane yield the transverse pulse properties.^16^

For the temporal characterization of ultrashort electron pulses, we perform electron-light cross-correlation measurements utilizing the inelastic scattering of free electrons at momentum-broadened femtosecond light fields.^69–71^ From the dependence of the electron energy spectra on the electron-light delay, we extract the electron pulse duration and chirp.

## III. COULOMB INTERACTIONS CLOSE TO THE EMITTER TIP

During illumination with the 400-nm laser pulse, photoelectrons are generated at the tip apex with a distribution of kinetic energies, emission directions, and sites. The beam structure and its propagation can be described by an evolving probability distribution in phase space spanned by the spatial and momentum electron coordinates. For close-to paraxial beam propagation, the phase space can be separated into transverse [Fig. 1(d)] and longitudinal sub-spaces [Fig. 1(c)], spanned by corresponding spatial coordinates as well as angular and energy coordinates.^32^

The initially populated region in phase space is defined by the spread in photoemission momenta and positions. In the space-charge-free regime, the occupied phase-space volume stays constant during free-space propagation or under pulse manipulation by conservative fields^72^ and thus determines the minimal focus size of the pulsed electron beam and its minimum temporal width. For higher bunch charges, space-charge effects within the electron pulse result in linear and non-linear distortions of the phase space density and additional irreversible blurring due to stochastic Coulomb interactions.^32,56,73,74^ For quantifying the occupied phase-space area, the root-mean-square (rms) emittance^32^ was introduced—a quantity closely related to the beam-quality parameter in optics^75^ and to the relative spatial and temporal coherence lengths.^16^

## IV. TRANSVERSE BEAM PROPERTIES AND SPECTRAL WIDTH OF ELECTRON PULSES

**FIG. 2.**
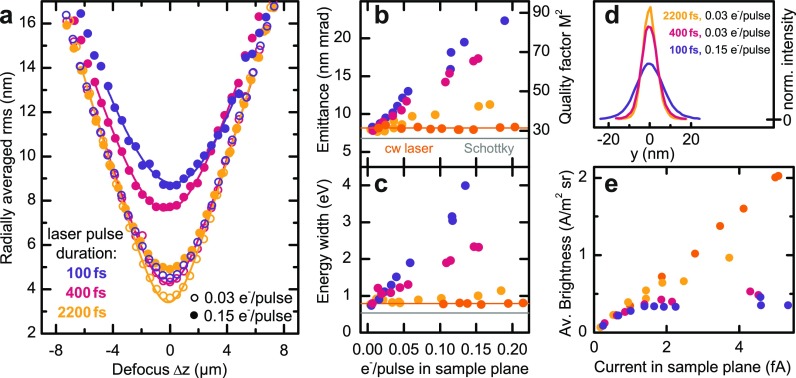
Experimental beam properties of photoelectron pulses. (a) Exemplary beam caustics are plotted for three different 400-nm laser pulse durations and two different electron bunch charges, characterized by the number of electrons counted in the sample plane. The laser pulse duration is varied by dispersive broadening in a 10-cm BK7 or SF6 glass bar. The solid curves are adapted to the experimental data, considering the caustic behavior of an electron beam with a beam quality factor $M^{2}$ [cf. figure (b)]. (b) and (c) Transverse electron beam emittance and energy width depending on the electron pulse charge in the sample plane (estimated bunch charge at the emitter: up to 200 electrons per pulse). As a reference for a space-charge-free beam, the emittance and energy width of a continuous photoelectron beam and for continuous thermal electron emission are given (orange symbols and gray line, respectively). (d) Focal spot profiles of the electron pulses for selected initial pulse durations and bunch charges exhibiting a close-to Gaussian shape. (e) Electron beam brightness depending on beam current shows a linear scaling in the low-charge regime and saturation at higher bunch charges. The saturation level depends on the initial pulse duration. An equal color coding for the initial pulse durations is used in all panels.

In characterizing the transverse electron beam properties, beam caustics are measured for different numbers of electrons per pulse and laser pulse durations. In Fig. 2(a), the rms electron spot radii, $\sigma_{r}$, are plotted as a function of defocus. We note that for the chosen gun settings, 0.15 electrons at the sample position correspond to about 10–100 photoemitted electrons at the tip. Larger beam currents (at reduced coherence) can be obtained for different gun settings optimized for an enhanced transmission ratio.^16^ The observed minimum beam sizes are significantly affected by Coulomb interactions, increasing from about 3 nm in the low-current/long-pulse regime (open yellow circles) to above 9 nm for larger bunch charges and short initial pulse durations (blue solid circles). The effect of space-charge induced broadening on the electron focal spot profiles is directly visible in the spot cross-sections [Fig. 2(d)].

For quantitatively assessing the beam properties, we extract the beam emittances, given by $\epsilon_{n,\text{rms},r}=\beta \gamma \sigma_{r}\sigma_{\alpha }$ with the relativistic Lorentz factor γ and $\beta =v_{e}/c$ (with $v_{e}$ and c being the electron and light velocities in vacuum, respectively). The corresponding angular spread of the beam, $\sigma_{\alpha }=4.4\;\text{mrad}$, is determined in the far-field and stays constant for the range of pulse parameters studied here.

The resulting emittance values are plotted in Fig. 2(b), exhibiting an approximately linear growth with increasing electron pulse charge. Larger initial pulse durations result in less increase in the emittance, signifying weaker Coulomb interactions. As a reference, we also determined the beam emittance generated by a continuous-wave photoemission laser (405-nm wavelength, average optical power varied between 0.2 mW and 3.5 mW). The mean emittance value (orange line) coincides with the value for pulsed electron beams in the low-charge limit. For comparison with transverse beam properties in light optics, we also show the extracted beam quality factor $M^{2}=\epsilon /\epsilon_{q}$ [Fig. 2(b), right axis].^16^ The quantity $\epsilon_{q}=\hbar /(2m_{e}c)$ is the minimum emittance for a fully coherent beam, as obtained from the Heisenberg uncertainty principle for the product $\sigma_{r}\sigma_{\alpha }$. Furthermore, utilizing the corresponding $M^{2}$ values and considering Gaussian-shaped beams, the caustic behavior of the electron pulses is well reproduced over the whole defocus range [solid curves in Fig. 2(a)].

Heating the tip to induce conventional Schottky emission, the caustic of the continuous electron beam was characterized. We obtain an emittance of 6.8 nm mrad (gray line), comparable to the results for low-current photoelectron pulses.

At the focal plane (Δz=0), the electron beam profiles remain Gaussian in shape, indicating that spherical aberrations of the objective lens (spherical aberration constant $C_{s}=1.4\;\text{mm}$) are negligible, and no significant nonlinear beam distortions are introduced by space-charge interactions within the pulse. Notably, within the experimental resolution, no shift in the longitudinal position of the focal plane is observed for higher pulse charges, i.e., the minimum spot diameter occurs at Δz=0.  This observation indicates the absence of considerable space-charge induced linear distortions in the transverse phase space.

The averaged beam brightness, as shown in Fig. 2(e), saturates at larger bunch charges due to Coulomb-induced emittance growth. Larger average brightness values are achievable for temporally stretched photoemission pulses (yellow dots), allowing for a tailoring of the photoemission parameters to the required beam brightness in ultrafast electron imaging experiments. As a limiting case, the continuous photoelectron beam exhibits no emittance growth in the utilized current range, resulting in a linear scaling of the beam brightness with the beam current.

Space-charge effects on the pulses' longitudinal phase-space structure are characterized by considering the width of the electron energy distribution. Similar to the emittance dependencies, we observe a linear increase in the spectral width with bunch charge and weaker space-charge effects for longer initial pulse durations [Fig. 2(c)]. For continuous laser illumination, the spectral bandwidth is set by the initial energy width after photoemission and the instrumental energy resolution.

## V. TEMPORAL STRUCTURE OF HIGH-CHARGE ELECTRON PULSES

**FIG. 3.**
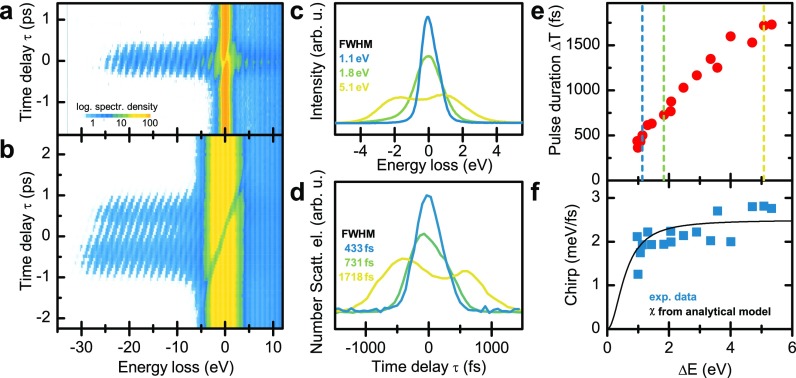
Longitudinal electron pulse characterization. (a) and (b) Energy spectra depending on the time delay between the excitation of an optical field (50-fs optical pulse duration, electron-light interaction facilitated by reflection off a thin silicon membrane) and the arrival time of the electron pulses (photoemission laser pulse duration: 100 fs). Photon sidebands on the energy-gain and -loss side are formed during temporal overlap, yielding an electron-light cross correlation. For low-charge electron pulses (a), short cross-correlation times are observed, substantially broadening for higher bunch charges (b). Color scale is chosen to highlight the temporal width of photon-sideband intensity on the gain-side of the spectra. (c) and (d) Space-charge induced spectral and temporal broadening is observed in the electron energy spectra (c) and temporal electron pulse profiles [(d) extracted from the delay-dependent intensity of higher order photon sidebands]. (e) Electron pulse duration (FWHM) scales linearly with its energy width. Dotted lines: position of spectral and temporal profiles shown in (c) and (d), respectively. (f) Electron pulse chirp (blue symbols) is derived from the inclination of the photon sidebands. The experimental data are well described by an analytical model (black line) considering an energy-independent shear amplitude due to pulse propagation and a space-charge-induced spectral broadening close to the emitter tip.

Space-charge broadening in the longitudinal direction results in an increase in the electron pulse duration. For quantitative characterization of the temporal electron pulse structure, we spatio-temporally overlap the electron pulses with an optical field driven by ultrashort laser pulses (800-nm central wavelength, 50-fs pulse duration, $10-\text{mJ}/cm^{2}$ fluence), reflected off a single-crystalline silicon membrane (35-nm thickness). For electrons arriving at the membrane while the transient optical field is present, inelastic electron-light scattering yields photon sidebands (spaced by the incident photon energy) in the electron energy spectra, as shown in Figs. 3(a) and 3(b). Due to the short pulse duration of the optical field, the temporal delay range τ over which higher-order sidebands are observed corresponds to the electron pulse duration.

Exemplarily, two spectro-temporal maps are chosen to demonstrate the influence of the number of electrons per pulse [Figs. 3(a) and 3(b)]. The corresponding electron energy spectra are shown in Fig. 3(c). In the few-electron regime [Fig. 3(a)], photon sidebands are only visible in an interval of a few hundred femtoseconds. For higher bunch charges, besides the spectral broadening discussed above, photon sidebands appear over a 2-ps delay range, signifying considerable temporal pulse broadening. Integration of the gain-scattered electrons over the delay time τ yields a quantitative measurement of the temporal profile of the electron pulses [Fig. 3(d)]. The extracted pulse durations (FWHM) are plotted in Fig. 3(e) as a function of the space-charge-induced spectral broadening. Between the shortest value of 350 fs for an energy width of 0.9 eV and the longest of 1700 fs for 5.3 eV, the pulse duration depends slightly sub-linearly on the imprinted energy width.

Furthermore, a pronounced chirp of the pulses is observed, as seen in Figs. 3(a) and 3(b). In particular, space-charge effects lead to an acceleration/deceleration of the electrons at the leading/trailing edge. Thereby, the electron's energy and its longitudinal position within the bunch are strongly correlated, providing for a direct mapping between the spectral and temporal profiles [Figs. 3(c) and 3(d)]. The chirp, plotted in Fig. 3(f), is quantitatively extracted by performing a Fourier analysis of the spectral sidebands (on the gain side) and analyzing the phase of frequency components corresponding to the sideband periodicity.

## VI. SIMULATION

For the numerical simulation of the transverse and longitudinal electron beam properties in the space-charge regime, we consider a simplified electron-optical geometry consisting of a spherical photocathode and an extractor electrode [Fig. 1(b)]. In the simulation, photoelectrons are randomly generated according to a homogeneous probability density at the surface of a spherical tip apex (240 nm tip radius, π/2 opening angle of emission surface, isotropic photoemission direction). Geometric dimensions of the model are chosen such that the emitting area and the distance between the tip and the extractor ($d_{\text{tip}-\text{ext}}=350\;\mu m$) resemble the experimental conditions. The electron pulse is propagated from the emitter to the extractor employing a Verlet algorithm,^76^ considering the interaction with the external electrostatic field, intrapulse Coulomb forces, and contributions from image charges at the emitter.

The influence of Coulomb interactions on the transverse and longitudinal properties of the electron pulses is investigated by varying the number of electrons per pulse for a set of initial pulse durations. To properly account for the beam apertures in the experiment, we select the central section of the simulated electron bunch for the beam analysis. The acceptance angle 2θ of the effective aperture (172 mrad) and the initial energy distribution after photoemission (0.9 eV) were adapted to the experimental emittance and the spectral width in the space-charge-free regime. The aperture size provides for a transmission ratio comparable to the beam limiting aperture (opening angle 200 mrad) in trajectory simulations within the non-spherical field-distribution of the suppressor-extractor geometry. We note that for our experimental conditions, the transmission ratio does not depend on the initial electron bunch charge and duration, so that a linear scaling between the photoemitted electron current and the current in the sample plane can be applied.

**FIG. 4.**
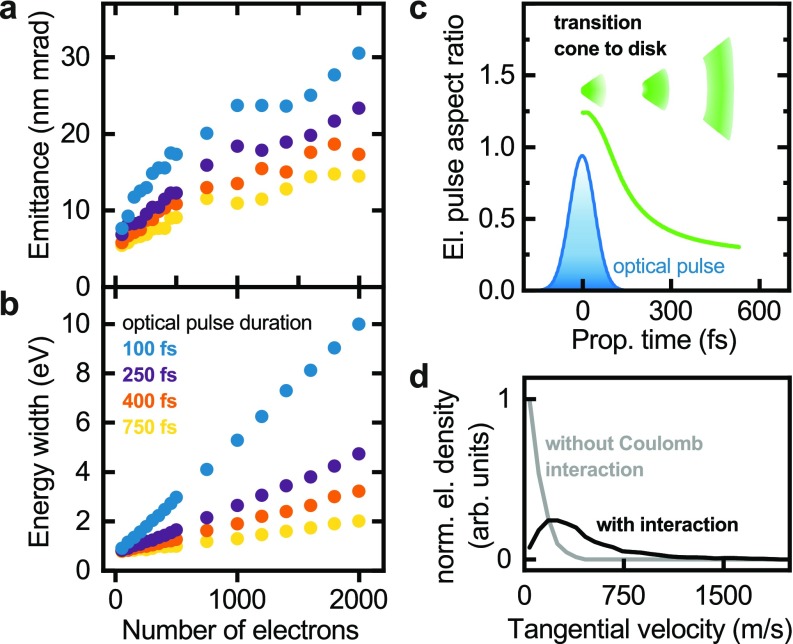
Simulated electron pulse properties. (a) and (b) Transverse electron beam emittance and energy width depending on the number of emitted electrons and the initial pulse durations. A linear scaling with the charge density is found. (c) Aspect ratio (longitudinal relative to transverse pulse width) of the electron pulse depending on the delay after arrival of the photoemission laser pulse at the tip apex. After photoemission, the pulse evolves from a conical to a disk-like shape. Blue curve: intensity profile of the photoemission laser. (d) Distribution of tangential electron velocities (i.e., velocity components perpendicular to the radius vector from the center of the spherical emitter apex) at a radial distance of 315 *μ*m from the emitter for the space-charge-free regime (gray curve) and with Coulomb interactions (black curve, considering 2000 electrons/pulse and an initial pulse duration of 100 fs).

The beam emittances and energy widths of the electron pulses are shown in Figs. 4(a) and 4(b), respectively. In close correspondence to the experimental results, a linear scaling of both properties with the bunch charge is found. In addition, longer initial pulse durations (i.e., for stretched laser pulses) largely reduce space-charge effects at a given bunch charge. The overall magnitude of Coulomb-induced emittance-growth for a given spectral broadening reproduces the experimental findings. Fully quantitative agreement would require detailed knowledge on the in-operando emitter tip shape and the local photoemission probability on its surface.

## VII. DISCUSSION

At tip-shaped photocathodes, electrons are accelerated by large electrostatic extraction fields on the order of 10^8^–10^9^ V/m and form a strongly diverging beam, with the beam eccentricity^73^ changing from a conical to a disk-like shape [Fig. 4(c) and Movie in the supplementary material]. Therefore, the initially high charge density (around $50\;{\text{e}}^{-}/{\mu m}^{3}$) around the emitter apex quickly disperses, and the emitted electrons experience considerable Coulomb forces for a short time only. The Coulomb effects observed in the sample plane are largely accumulated within the first few micrometers after photoemission. For example, about 80% of the final spectral width is gained within the first 4 *μ*m of the propagation distance, for an electron bunch charge of 150 electrons and 100 fs initial pulse duration. After the beam-defining aperture in the focus electrode, the pulse contains less than one electron on average, so that intra-pulse Coulomb interactions, e.g., at cross-overs, are negligible. We note that such a combination of high-charge density at the tip and subsequent space-charge-free propagation drastically differs from the regimes studied for many-electron femtosecond pulses generated from flat photocathodes, often utilized in ultrafast electron diffraction studies^77^ or in dynamic TEM with nanosecond temporal resolution.^78^

Using the quasi-instantaneous space-charge-induced electron broadening at the tip, we arrive at an analytical model which links the energy width to the pulse duration and chirp at the sample position. In particular, we consider the propagation time $T=\int dz\;{\left[2/m_{e}\left(E_{0}+e\phi (z)\right)\right]}^{-1/2}$ of electrons with an initial energy $E_{0}\;$ along the optical axis, where $m_{e}$ and e are the electron mass and its charge and ϕ(z) is the on-axis potential. To properly include the static field enhancement at the tip, we choose a parabolic electrostatic potential^79^ between the tip and the extractor and plate-capacitor-like geometries for the subsequent acceleration stages. From the slope of T(E), we evaluate the shearing angle of the longitudinal phase space distribution due to propagation from the source to the sample, yielding a shear of ${\frac{\partial T}{\partial E}=s}_{E}=480\;\text{fs}/\text{eV}$.

Whereas the space-charge induced energy broadening occurs within 4 *μ*m after photoemission (within approximately 200 fs after photoemission), the translation of this broadened energy distribution into a temporal width is mainly accumulated during the first two acceleration stages, i.e., in-between the photocathode, extractor, and focus anodes (shear contributions of 46% and 48%, respectively). Only a minor shear results from the further propagation behind the focus anode.

In the inelastic electron-light scattering experiments (Fig. 3), the spectral chirp of the electron pulses is evident from the change of the mean electron energy with time. In general, the relationship between the shear in longitudinal phase space and the observed spectral chirp depends on the shape of the electron distribution function.^70^ Considering a sheared Gaussian distribution $f_{s}\left(t,E\right)=Ne^{-\frac{{\left(t-s_{E}E\right)}^{2}}{2\sigma_{t}^{2}}}e^{-\frac{E^{2}}{2\sigma_{E}^{2}}}$, in which $\sigma_{E}$ is the electron energy width, $\sigma_{t}$ the initial pulse duration, and N the normalization constant, we obtain the chirp $\chi =\;\frac{\partial \left\langle E\right\rangle }{\partial t}=\frac{\sigma_{E}^{2}s_{E}}{\sigma_{E}^{2}s_{E}^{2}+\sigma_{t}^{2}}$. For $\sigma_{t}=100\;\text{fs}$ and $s_{E}=400\;\text{fs}/\text{eV}$, the experimental data are well reproduced [Fig. 3(f), black curve]. We note that for distributions with $\sigma_{E}s_{E}>\sigma_{t}$, i.e., in the limit of large shear or spectral width, the electron pulse chirp is given by the inverse shear, $\chi =\;\frac{1}{s_{E}}\;$. In this limit, the electron pulse duration at the sample $\Delta T=s_{E}\;\Delta E$ is directly linked to the spectral width. The shear constant $s_{E}$ is governed by the chosen electrostatic potentials in the emitter gun, providing an approach to monitor pulse durations and to minimize the temporal spread for a given space-charge-induced spectral broadening.

Different from the reversible distortion of the electron distribution in the longitudinal direction, transverse electron pulse properties are affected by an emittance growth. In order to gain a microscopic picture of the underlying processes from the simulation results, we consider the electron velocities at a radial distance of 315 *μ*m from the emitter. In a spherical coordinate system with its origin at the center of the emitter half sphere, the tangential velocity component, i.e., perpendicular to the radial vector, is a measure of the deviation from a fully coherent beam. The velocity distribution in the space-charge-free case [Fig. 4(d), gray curve] is related to the initial photoemission velocity. Space-charge interactions result in a pronounced broadening of the distribution of tangential velocities [Fig. 4(d), black curve], signifying an increase in the back-projected electron source size and thus an increased beam emittance.

## VIII. CONCLUSION

In conclusion, we systematically evaluated the properties of a laser-driven Schottky field emitter for ultrafast transmission electron microscopy, operated in a regime of a high-density of photoemitted electron bunches. The observed pulse properties are well described by a simplified model of dense electron pulses propagating in a high and strongly divergent extraction field. Electron pulse properties in the longitudinal and transverse directions are governed by reversible space-charge induced spectral broadening with subsequent pulse shearing and stochastic emittance growth, respectively, predominantly occurring within a distance of a few micrometers from the emitter. Large extraction fields at the emitter apex allow for dense nanoscale pulses with only moderate Coulomb-induced beam deterioration. The presented theoretical framework and demonstrated control of electron pulse properties generally apply for nanoscale photocathodes in similar gun geometries and enable the generation of tailored electron pulses adapted to the specific requirements of ultrafast electron imaging, diffraction, and spectroscopy experiments.

## SUPPLEMENTARY MATERIAL

See supplementary material for details on the space-charge free electron pulse propagation as obtained from numerical simulations (video). The electron pulse strongly diverges due to the high extraction field and changes its geometrical eccentricity from a conical to a disk-like shape.
